# Metabolic reprogramming in prostate cancer

**DOI:** 10.1038/s41416-021-01435-5

**Published:** 2021-07-14

**Authors:** Fahim Ahmad, Murali Krishna Cherukuri, Peter L. Choyke

**Affiliations:** 1 Molecular Imaging Branch, National Cancer Institute, National Institutes of Health, Bethesda, MD USA; 2 Radiation Biology Branch, National Cancer Institute, National Institutes of Health, Bethesda, MD USA

**Keywords:** Cancer imaging, Cancer metabolism

## Abstract

Although low risk localised prostate cancer has an excellent prognosis owing to effective treatments, such as surgery, radiation, cryosurgery and hormone therapy, metastatic prostate cancer remains incurable. Existing therapeutic regimens prolong life; however, they are beset by problems of resistance, resulting in poor outcomes. Treatment resistance arises primarily from tumour heterogeneity, altered genetic signatures and metabolic reprogramming, all of which enable the tumour to serially adapt to drugs during the course of treatment. In this review, we focus on alterations in the metabolism of prostate cancer, including genetic signatures and molecular pathways associated with metabolic reprogramming. Advances in our understanding of prostate cancer metabolism might help to explain many of the adaptive responses that are induced by therapy, which might, in turn, lead to the attainment of more durable therapeutic responses.

## Background

**Fig. 1:**
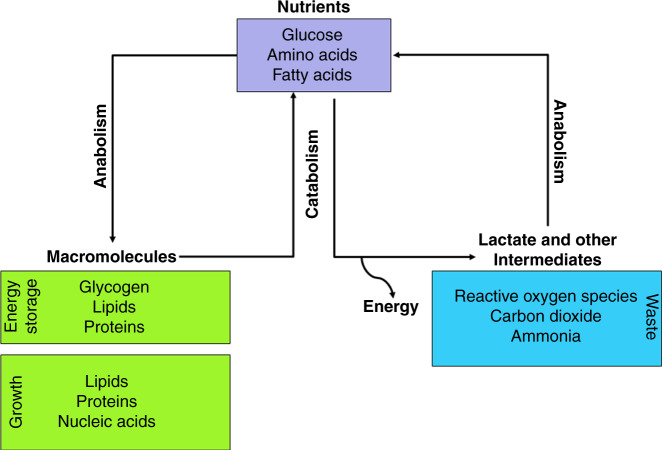
Core metabolic pathways. Catabolic pathways—for example glycolysis, oxidative phosphorylation via tricarboxylic acid cycle (TCA) and lipogenesis—involve the breakdown of major nutrients (glucose, amino acids and fatty acids) to generate energy, which is either stored for later use or released as heat. Anabolic pathways then build macromolecules out of the products of catabolism, which are building blocks for cell structures and help to maintain the cell. Cells also produce lactate, ammonia, carbon dioxide and reactive oxygen species (ROS) as by-products of the metabolic breakdown of sugars, fats and proteins. Emerging studies have revealed a functional role for many of these metabolic by-products. For many years, lactate was seen as the metabolic waste product of glycolytic metabolism; however, new roles for lactate in the tumour microenvironment as a metabolic fuel, modulator of extracellular pH or as a signalling molecule have emerged. Ammonia, generated as a by-product of proteins that are broken into amino acids by amino acid lyases and nucleotide deaminases, is toxic at high concentrations and, thus, gets further converted into urea. ROS, mainly generated through NADPH oxidase (NOX4) and through electron leak from electron transport chain (ETC) complexes, are potent mitogens that promote proliferation, differentiation and migration.

Cellular metabolism involves complex biochemical processes through which specific nutrients are consumed. Carbohydrates, fatty acids and amino acids are the main source of nutrients for energy homeostasis and macromolecular synthesis. They are the main constituent of core metabolic pathways that can be classified as anabolic, catabolic or waste producing (Fig. 1) and that mediate processes such as glycolysis, oxidative phosphorylation via the tricarboxylic acid (TCA) cycle, glycogenolysis, lipogenesis and the urea cycle.

Unlike normal cells, cancer cells rewire their cellular metabolism to promote growth and survival and thus have different nutritional requirements, and many different cancer types exhibit similar metabolic alterations.^1,2^ The Warburg effect, for example, is a change in the metabolism of most cancer cells that enables them to convert glucose into lactate, even in the presence of abundant oxygen—in a process known as aerobic glycolysis.^3^ This change was originally thought to be due to defective oxidation caused by mitochondrial dysfunction, but has now been partly explained biologically^4,5^: the rapid uptake and metabolism of glucose allows cells to feed several non-mitochondrial pathways, such as the pentose phosphate pathway, which produces ribose for nucleotides and NADPH for reductive biosynthesis, and the hexosamine pathway, which is required for protein glycosylation and glycerol synthesis for production of complex lipids. The Warburg effect might support anabolic metabolism indirectly while maintaining large pools of glycolytic intermediates that favour engagement of the pentose phosphate pathway and other biosynthetic pathways inside the cell.^6^ Although the exact mechanism(s) of altered metabolism and its effect on cancer behaviour is unknown, an increased awareness of the dependencies of cancer cells on specific metabolic pathways and the potential for therapeutically exploiting these dependencies have led to an interest in better understanding the underlying processes.^7^

Several tools have been designed to assess metabolism without disturbing the system. Advances in nuclear magnetic resonance and mass spectroscopy have helped to quantify the carbon influx of the central metabolic pathways (e.g. TCA cycle, glycolysis and pentose phosphate pathway) in mammalian systems, and this approach has been instrumental in demonstrating that the metabolism of a tumour depends on factors such as the tissue of origin, the tumour microenvironment (TME), the level of hypoxia, and so on. Furthermore, tumour cells appear to turn on different programs of metabolic pathways to generate ATP, proteins, nucleotides and lipids for cellular proliferation compared with normal cells.^8^

This review will explore important conceptual and technological advances in cancer metabolism with the goal of motivating the ongoing research in tumour metabolism. It further emphasises the critical roles of nutrient availability, oxygen concentration, genetic signatures, tissue origin and the TME in determining the metabolic reprogramming that is necessary to sustain tumour growth. Focussing on prostate cancer, the unique metabolic program driven by the androgen receptor (AR) and the role of this program in fuelling oncogenic growth of prostate cancer are also described. Finally, we highlight possible future avenues of research needed to close current knowledge gaps.

## Factors affecting metabolism in cancer cells

Cancer cells exhibit an altered metabolism to fulfil the demands of increased growth and proliferation. This alteration is, in part, orchestrated by the genetic changes that govern tumorigenesis,^2^ such as the activation of oncogenes and/or the loss of tumour suppressor genes, and is further shaped by environmental factors, such as nutrient availability and oxygen concentration.^9^ Tumours predominantly exhibit heterogenous metabolic reprogramming and different tumours employ a varied array of metabolic pathways to aid anabolic requirements during growth and proliferation. Thus, a detailed understanding of metabolic rewiring is essential to explain the fundamental mechanisms of tumorigenesis.

### Genetic changes

Major shifts are seen in amino acid, nucleotide and lipid metabolic pathways as a result of changes in the genetic landscape in cancer cells. The amplification and/or mutation of genes implicated in growth factor signalling is relatively common in cancer cells. For instance, the epidermal growth factor receptor (EGFR) tyrosine kinase receptor activates downstream effectors including phosphatidylinositol 3-kinase (PI3K) and AKT signalling.^10–14^ Further activation of the PI3K–AKT pathway reprogrammes cellular metabolism by augmenting the activity of nutrient transporters and metabolic enzymes, thereby supporting the anabolic demands of aberrantly growing cells.^10–14^ Genes such as *KRAS* and *c-Myc* are commonly amplified/mutated in tumours and frequently influence nutrient uptake and utilisation. In some cases, the same genetic alterations that regulate glycolysis can also co-ordinately regulate other metabolic processes. For example, the EGFR oncogenic variant EGFRvIII co-ordinately controls intratumour cholesterol levels and regulates fatty acid synthesis.^15–18^ A link between c-Myc and mTROC-1 and amino acid regulation and nucleotide biosynthesis has also been observed.^18–21^ In other cases, mutations often cause a loss of function, which promotes the use of alternative metabolic pathways. Thus, genetic signatures can strongly influence metabolism.

## Environmental factors

Cancer cells alter the chemical composition of extracellular milieu, which exerts pleiotropic effects on both the normal cells residing in the vicinity of the tumour and also the extracellular matrix.^22^ Reciprocally, the microenvironment affects the metabolism and signalling responses of cancer cells. Tumours are often faced with limited nutrient and oxygen supply and develop various nutrient scavenging strategies to circumvent this limitation. Furthermore, hypoxia impedes the ability of cells to carry out oxidative phosphorylation and other reactions that require oxygen, and disrupts the redox balance, affecting cellular signalling and transcriptional programs.^2^ Overall, the reciprocal interactions between the tumour and their microenvironment impose a selective pressure that further shapes the cellular metabolism.

### Nutrient supply

During the early stage of tumorigenesis, tumour cells are in close contact with the normal vasculature and are therefore supplied with ample nutrients (and oxygen). However, once these cells outgrow the normal vasculature, they must alter their metabolism to adjust to lower nutrient concentrations.^23^ This process depends on an intrinsic nutrient-sensing mechanism, which triggers nutrient-responsive transcription factors and signalling pathways. Hypoxia-inducible factor (HIFs), sterol regulatory-element binding transcription factor (SREBFs) and activating transcription factor 4 (ATF4) are among the transcription factors that aid tumour cells in adapting to fluctuations in nutrient availability. The induction of HIF1-α and ATF4 in response to limiting nutrient conditions, results in the upregulation of glucose and amino acid transporters, which helps cancer cells to compete for various nutrients^24^; HIF1-α also induces the expression of glycolytic enzymes, thereby facilitating sustained ATP production.^25^ Oncogenic driver mutations, such as those in c-Myc, can result in the aberrant activation of mammalian target of rapamycin complex (mTORC)-1, which promotes nutrient uptake and the anabolic conversion necessary for cell growth and division.^26^

**Fig. 2:**
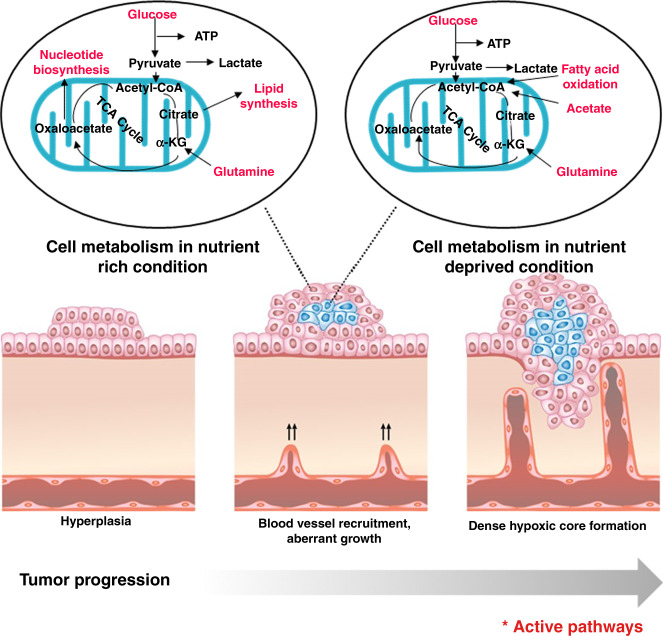
Metabolic pathways adopted by cancer cells under varying nutrient conditions. The proximity to the vasculature determines the accessibility of cells within solid tumours to nutrients. Cells that are near to the vasculature acquire nutrients and oxygen to fuel anabolic pathways, but cells that are further away have decreased accessibility to nutrients and oxygen and might employ alternative pathways, including oxidation of fatty acids, to meet the bioenergetic demands of proliferation and growth.

**Fig. 3:**
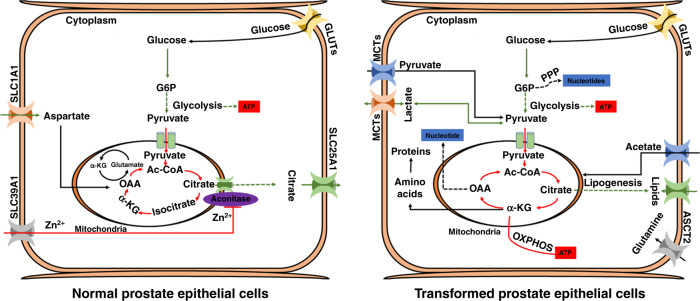
Metabolism of normal versus transformed prostate epithelial cells. Normal prostate epithelial cells assimilate glucose and aspartate to synthesise and secrete citrate. Androgen-receptor (AR)-mediated metabolic reprogramming drives enhanced oxidative phosphorylation and lipogenesis inside the transformed prostate epithelium. Normal prostate epithelial cells produce most of their ATP via glycolysis, whereas oxidative phosphorylation remains the main source of ATP generation in early prostate adenocarcinoma. Highlighted red and green arrows represent metabolic pathways that are important in normal and transformed prostate epithelial cells. Solid arrows represent single metabolic steps and dashed arrows represent simplified multistep processes. SCL39A1, SLC25A1, SLC1A1, GLUTs and MCTs represent zinc, citrate, aspartate, glucose, pyruvate and lactate transporters, respectively.

Angiogenesis is another important adaptation that facilitates greater nutrient delivery, but this is not always successful. When tumour tissues require fuel (nutrients and oxygen), angiogenesis is stimulated. However, upregulation of angiogenic activator is not solely sufficient, negative inhibitors of vessel growth also need to be down regulated for angiogenesis^27^ (Fig. 2). The uptake of glucose, the primary energy source of cells, can be measured using 18F-fluorodeoxyglucose (FDG, a radiolabelled glucose analogue). Many primary tumours, including primary prostate carcinoma, show little or no FDG uptake,^28^ which suggests that metabolic substrates other than glucose might be required to meet the anabolic demands. Clinical imaging studies clearly demonstrate evidence of the uptake of exogenous acetate^29^ and pyruvate^30^ in prostate cancers, both of which can be further incorporated directly into the TCA cycle or directed to lipogenic metabolism (Fig. 3).

### Oxygen supply

Oxygen is crucial for supporting cellular bioenergetics and other specific biochemical reactions. In 1977, Thomlinson and Gray revealed that tumours display a gradient of oxygen concentration, with the tumour core being the most oxygen-limited region.^31^ Whereas normal cells succumb to hypoxic conditions, cancer cells can undergo transcriptomic changes that enable them to survive and adapt to this hostile microenvironment. As their name suggests, HIF proteins are induced in response to low oxygen concentrations, and increased levels have been linked to malignancy and poor prognosis in various cancers including prostate cancer.^32^ As well as mediating an increase in the levels of glycolytic enzymes,^33^ the major isoform, HIF1-α, induces angiogenesis by secreting factors such as vascular endothelial growth factor (VEGF), which promotes neovascularisation.^34^ Another adaptation to hypoxic conditions involves the enhanced utilisation of glutamine, which sustains part of the TCA cycle and promotes lipid synthesis.^35^ Hypoxia, therefore, is an important factor in metabolic reprogramming.

### Tissue origin and TME

Different tissues provide distinct environments for tumours, and thus the context-dependent nutrients, extracellular matrix components and interactions with stromal cells can differently influence metabolic pathways. An analysis across 20 different cancer types indicates that cancers undergo a tissue-specific rewiring of metabolic genes.^36^ Activation of nucleotide synthesis and inhibition of mitochondrial metabolism are the main features of convergent metabolic landscape of cancer. Furthermore, downregulation of oxidative phosphorylation correlates with poor clinical outcome across several cancer types and it is associated with the presence of epithelial-to-mesenchymal (EMT) signature.^36^ Hensley et al. also revealed the existence of regional differences in tumour nutrient utilisation by using intraoperative ^13^C-glucose infusions in human non-small cell lung cancer (NSCLC). The study revealed that less-well-perfused regions of human NSCLC tumours had elevated levels of glucose oxidation while highly perfused regions used non-glucose nutrients for oxidation,^37^ highlighting the importance of the microenvironment in metabolic dependency and the role of metabolic flexibility both among patients and within individual tumours. For metastatic tumours, the metabolic dependency on glycolysis or oxidative phosphorylation is often determined by the site of metastasis.^38^

Metabolic heterogeneity within a tumour is the result of differential exposure of various regions to distinct microenvironments. The clinical and morphological heterogeneity of prostate cancer alongside a high level of genetic diversity makes this cancer type a paradigm for genetic and metabolic heterogeneity in research settings, and the remainder of the article will focus on this cancer type.

## Metabolism in the benign prostate gland

The primary function of the prostate gland is to produce and secrete prostatic fluid, which lubricates, nourishes and protects sperm. This secretory activity is mainly dependent on hormone signalling mediated through the androgen receptor (AR), a nuclear hormone receptor transcription factor.^39,40^ To better understand metabolic alterations in prostate cancer cells, it is important to be familiar with the metabolism of benign prostate cells (Fig. 3). Metabolic pathways have been found to be well regulated and highly specialised inside healthy prostate epithelial cells. Prostate epithelial cells, of which there are three different types (luminal, basal and neuroendocrine), comprise about 70% of the gland and are the main functional component. Specialised acinar epithelial cells, which line the prostate and give rise to the most common type of prostate cancer, acinar adenocarcinoma, accumulate large reservoirs of zinc. Zinc specifically inhibits the action of mitochondrial aconitase (ACO2), the enzyme that catalyses the oxidation of citrate,^41^ which ultimately prevents citrate from entering and, thus disrupting, the TCA cycle; instead, citrate (synthesised from aspartate and glucose) is secreted as an important component of prostatic fluid. Consequently, to meet the energy requirements for survival and propagation, the citrate-producing prostate cells probably adopt alternative metabolic pathways and restrict other nonessential activities. For instance, studies suggest that citrate-producing prostate cells exhibit high levels of aerobic glycolysis.^39^ Unfortunately, extensive knowledge of these alternative and additional metabolic pathways in the prostate is lacking and needs further investigation.

## Prostate cancer metabolism

The unique, citrate-orientated metabolism of normal prostate tissue suggests that tumours arising from peripheral prostatic epithelium might also exhibit unique metabolic properties (Fig. 3). Primary prostate tumours seem to favour enhanced oxidative phosphorylation but limited glycolysis. Lipogenesis in the form of fatty acid synthesis also seems to be an early event in prostate tumorigenesis and is associated with progression of the disease.^42,43^ Increased glycolysis becomes a characteristic of advanced castrate resistant prostate cancer. Amino acid metabolism plays a crucial role in prostate cancer progression, including maintaining the amino acid pool, which is required as a building block, conversion to glucose, lipids and precursors of nitrogen containing metabolites like purines and pyrimidines for nucleic acid synthesis.^44^ Unlike other solid tumours the metabolism and biosynthesis of amino acids in prostate cancer is focused particularly on anaplerosis more than on energy production. Several potentially useful deviation in amino acid patterns have been discovered in malignant vs non-malignant prostate cancer cell lines and in urine of prostate cancer patients.^44^ Although considerable progress has been made, the molecular characterisation of prostate tumour metabolism is incomplete, and is likely to vary among tumours and even within individual lesions.

### AR-driven prostate cancer metabolism

AR signalling is crucial for the unique metabolic program of the prostatic epithelium and prostate adenocarcinoma. In healthy prostatic epithelial tissue, testosterone in the blood is converted by 5α-reductase to dihydrotestosterone (DHT), which binds to ARs in the cytoplasm of epithelial prostate cells. Once bound by DHT, AR transfers to the nucleus, where it can act as a transcription factor for numerous genes, such as KLK3 (PSA), KLK2 and NKX3-1.^45^ Testosterone also stimulates the production of citrate in prostate epithelial cells and transcriptionally through AR regulates the expression of the zinc transporter SLC39A1^46^ and the aspartate transporter SLC1A1,^47^ which facilitates citrate synthesis.

In prostate tumours, AR-mediated metabolic reprogramming is responsible for a metabolic shift to oxidative phosphorylation coupled with a loss of zinc transporter during oncogenic transformation. Zinc-regulated, Iron-regulated transporter-like (ZIP-1, 2, 3 and 4) proteins are suggested to be the major zinc uptake transporter involved in extraction of zinc from the blood and encoded by various subfamilies of SLC39 transporter. Studies have shown a correlation between the expression of ZIP proteins with changing intracellular zinc concentration.^48,49^ The depletion of zinc prevents the inhibition of mitochondrial ACO2, thereby restoring the TCA cycle; accordingly, the levels of citrate as well as zinc are much lower in malignant prostate cancer cells than in normal prostate tissue. Swinnen et al. and Audet-Walsh et al. demonstrated that activation of AR drives lipogenesis and oxidative phosphorylation, thereby supporting cellular proliferation.^50,51^ AR also fuels proliferation via other central metabolic pathways, including glycolysis (through the induction of the glucose transporter GLUT1, hexokinase 1, 2 (HK1) and HK2), and the pentose phosphate pathway through glucose-6-phosphate dehydrogenase (G6PD)). Mechanistic evaluation in prostate cancer cell line models suggests that AR-mediated regulation of the pentose phosphate pathway occurs through upregulation of G6PD in response to mTOR,^52^ resulting in the production of nucleotide precursors for DNA synthesis and NADPH to sustain lipogenesis.^53^

The complexity of AR-dependent metabolic changes has made it difficult to completely molecular characterise prostate cancer metabolism. Later in the disease course, prostate cancer cells can become androgen independent and, eventually, even AR independent. The withdrawal of androgens in androgen-dependent prostate cancer results in a rapid and dramatic regression of prostate adenocarcinoma through apoptosis, indicating the critical role played by AR in sustaining cancer cells in this context.^54^ However, various lesions of prostate adenocarcinoma can withstand hormone withdrawal and are able to reactivate AR-driven signalling and metabolism even in the absence of androgens. Reactivation results in prostate adenocarcinoma hypermetabolism, growth and metastatic dissemination,^55^ eventually leading to androgen independence (also referred to as castration-resistant disease and de-differentiated prostate cancer).

### Neuroendocrine prostate cancer metabolism

Neuroendocrine prostate cancer (NEPC) comprises an aggressive form of the disease that can arise de novo but more often forms as a consequence of the selective pressure of androgen deprivation; NEPC tumours are notable for their diminished AR signalling, increased expression of neuroendocrine lineage markers including chromogranin-A, neuron-specific enolase and synaptophysin, and their pure/mixed small cell histology.^56^ Genetic alterations, such as the loss of *RB1* and *TP53* and amplification of *MYCN* and *AURKA*, are more prevalent in NEPC compared with prostate adenocarcinoma. Functional loss of *RB1* and *TP53* facilitated the activation of pluripotency networks through de-repression of the pluripotency transcription factor SOX2 as well as the epigenetic modifier, enhancer of zeste-homolog 2 (EZH2).^57,58^ Further, modulation of the epigenome is tightly coupled to metabolic reprogramming; several chemical modifiers of DNA and histones are intermediates of cellular metabolic pathways.^59^ For example, glycolysis generates pyruvate, which is the main substrate for acetyl-CoA—that plays the central role in coordinating the activity of histone acetyltransferase (HAT) enzymes. Elevated expression of histone lysine demethylase KDM8 in treatment induced NEPC tumours functions to reprogram metabolism towards aerobic glycolysis.^60^ Also, MYCN, that is involved in neuroendocrine lineage reprogramming leads to elevated histone acetylation and DNA accessibility by increasing the mitochondrial export of acetyl groups and also by upregulating the HAT-GCN5. The NEPC-specific upregulation of the metabolic enzyme phosphoglycerate dehydrogenase (PHGDH) was identified in 2019 while profiling adenocarcinoma and NEPC tumours.^61^ Upregulation of PHGDH is the first and rate-limiting step in the serine, glycine, one-carbon pathway (SGOCP). Increased SGOCP activity in prostate carcinoma is mainly fuelled by the loss of atypical protein kinase C (PKC) λ/ι, and promotes the biosynthesis of serine, which, in turn, sustains increased proliferation and epigenetic demands (e.g. methylation through increased intracellular S-adenosyl methionine) to favour cancer cell plasticity and NEPC.^61^ In NEPC, increased glycolysis along with enhanced glutamine uptake can further increase the production of pyruvate and acetyl-CoA.^62^ Furthermore, elevated glycolysis coupled with MCT-4-mediated lactic acid production/secretion is the most distinguishing and clinically relevant metabolic feature in NEPC.^63^ Together, these studies suggest that NEPC differentiation depends on metabolic reprogramming to fuel the epigenetic changes that are required for this lineage conversion. The identification of metabolic, and other, non-oncogenic, dependencies might reveal vulnerabilities that could potentially open therapeutic avenues for the treatment of this highly aggressive treatment-resistant cancer.

## Oxidative phosphorylation

In prostate cancer, AR signalling promotes TCA cycle functions, including amino acid production, oxidative phosphorylation and lipogenesis.^39,64^ Broad molecular characterisation studies involving high-throughput mass spectrometry found that prostate tumours had increased levels of ACO2, citrate synthase, fumarate hydratase, malate dehydrogenase-2 and oxoglutarate dehydrogenase,^65^ and metabolite analysis coupled with transcriptomic data revealed elevated levels of TCA cycle intermediates, such as malate, fumarate and succinate, in prostate tumours compared with adjacent normal tissues.^66^ These results indicate that prostate adenocarcinoma, at least in the initial stages, is more reliant on oxidative phosphorylation than aerobic glycolysis.^67^

### Targeting oxidative phosphorylation in prostate cancer cells

Oxidative phosphorylation fuels cellular proliferation as, beyond the production of ATP, it is also required for aspartate production, which, in turn, regulates nucleotide biosynthesis. Unlike other cells, however, prostate epithelial cells can efficiently import exogenous aspartate, which is converted to oxaloacetate before condensation with acetyl-CoA to synthesise citrate under normal conditions. Therefore, although prostate cancer might be reliant on oxidative phosphorylation,^68^ pharmacological inhibition might prove challenging if cells can circumvent this requirement by importing aspartate. Oxidative phosphorylation can also be compromised by restricting the supply of reducing equivalent in the electron transport chain (ETC) by disrupting the production of NADH, or by directly targeting components of the ETC. Targeting TCA cycle metabolism by compromising mitochondrial substrate trafficking might be an effective strategy. For example, rotenone and metformin (Table 1), widely recognised inhibitors of complex-I of the ETC, inhibit proliferation in several human cancer cell lines,^69,70^ including prostate cancer.^71^ PTEN-null cells are highly sensitive to inhibition of complex-I, as their glucose levels are known to be rapidly depleted when ETC is suppressed, which highlights the potential clinical utility of complex-I inhibition of ETC. In agreement with this prediction, the rotenone derivative deguelin suppressed tumour growth in a prostate cancer mouse model lacking both *PTEN* and *Trp53*.^72^ Overall, these data provide evidence that targeting oxidative phosphorylation might be an effective therapeutic approach for prostate cancer.

**Table. 1:** List of metabolic inhibitors targeting prostate cancer.

S. NO	Drug	Target	Mechanism of action
1	Metformin	AMPK, mTORC1, ETC complex 1	Inhibits tumour proliferation, confers survival benefit in retrospective prostate tumour cohort^135–137^
2	MSDC-0160	Mitochondrial pyruvate carrier	Decreases mitochondrial oxygen consumption, depletes TCA cycle intermediates in hormone-responsive and castration-resistant AR^+^ models of prostate cancer^39^
3	IACS-010759	ETC complex-I	Inhibits proliferation, depletes macromolecule pools and induces apoptosis in *PTEN*-null mouse models of prostate cancer^138^
4	Fatostatin	SREBP–SCAP	Inhibits lipogenesis, blocks tumour growth and metastatic spread in metastatic and non-metastatic autochthonous models of mouse prostate cancer^93^
5.	MT 63-78	AMPK	Inhibits cell proliferation, induces mitotic arrest and apoptosis; activates AMPK and suppresses lipogenesis in hormone-responsive and castration-resistant AR^+^ and AR^–^ models of prostate cancer^139^
6	IPI-9119	FASN	FASN inhibition through IP-9119 leads to reduced protein expression and decreased transcriptional activity of full-length AR as well as splice variant AR V7. Thus, antagonises growth through metabolic reprogramming in hormone-responsive and castration-resistant AR^+^ models of prostate cancer.^86^

*AMPK 5’* AMP-activated protein kinase, *AR* androgen receptor, *ETC* electron transport chain, *mTORC* mammalian target of rapamycin complex, *SREBP–SCAP* sterol regulatory-element binding protein–SREBP cleavage-activating protein.

## Lipid metabolism

Lipids, including fatty acids, phospholipids and cholesterol, play a crucial role in the progression of prostate cancer. Lipogenesis generates intracellular signalling molecules along with raw materials for the production of lipid bilayers, precursors for cholesterol biosynthesis to promote intratumoural androgen synthesis and biochemical energy through β-oxidation.^73–75^ The increased accumulation of cholesterol as cholesteryl esters stored in cytosolic lipid droplets^76^ in prostate cancer cells lacking PTEN supports the role of lipid metabolism in tumour growth. Furthermore, the use of PET agents such as ^11^C acetate and ^11^C choline has successfully demonstrated the role of lipid metabolism in prostate cancer, including metastatic disease.

### Fatty acids

Fatty acids have been observed to translocate from regional pelvic adipocytes to prostate cancer cells,^77^ and an increase in the de novo synthesis of fatty acids^78^ and phospholipids, without a corresponding accumulation of lipids, has been reported. The hypothesis that increased fatty acid oxidation acts as a source of energy^79^ is supported by the known overexpression of α-methylacyl-CoA racemase (AMACR) in prostate cancer.^80^ This enzyme, which functions independently of androgen-mediated signalling,^81^ is required for the oxidation of branched chain fatty acids. Thus, it can be surmised that a balance between lipid synthesis and oxidation exists, providing cells with both a source of lipids (e.g. phosphocholine for cell membrane synthesis) and energy for growth and survival.

Dysregulated de novo fatty acid synthesis frequently occurs in malignancy.^82^ Oncogenic signalling pathways such as PI3K/AKT and HER2^75^ often result in the increased expression of lipogenic enzymes in prostate cancer cells, and the increased expression of fatty acid synthetic enzymes is associated with the nuclear localisation of AKT.^83^ Oncomine^84^ exploration of prostate cancer databases has revealed the upregulation of several mRNAs that encode lipid metabolic enzymes, such as ATP citrate lyase (ACLY), acetyl-CoA carboxylase-α (ACACA), fatty acid synthase (FASN) and long chain fatty acyl-CoA synthetases-1, 3 and 5 (ACSL-1, ACSL-3, ACSL-5). The increased expression of transcription factors such as sterol regulatory binding transcription factor-1 (SREBF-1), which is responsible for regulating the expression of fatty acid and cholesterol synthesising enzymes, has also been reported.^85^ Metabolic reprogramming in hormone-responsive and castration-resistant AR^+^ models of prostate cancer is mediated by fatty acid synthase, and pharmacological inhibition of FASN with IP-9119 (Table 1) leads to the reduced expression of AR and a consequent decreased in its transcriptional activity, which ultimately compromises tumour growth.^86^

### Cholesterol

A functional relationship exists between cholesterol metabolism and prostate cancer progression. High circulating levels of cholesterol are positively associated with the development of prostate cancer,^87^ and cytosolic lipid droplets containing cholesteryl esters have been linked with prostate cancer aggressiveness.^76^ Accordingly, pharmacological inactivation of the cholesterol acyltransferase enzyme (acetyl-CoA) results in apoptosis accompanied by a decrease in cellular proliferation, migration and invasion.^88^ Activation of PI3K–AKT–mTOR as a consequence of the loss of PTEN is associated with the upregulation of SREBP and low-density lipoprotein (LDL) receptors, which induce the accumulation of cholesteryl esters.^89^ The coordinated, opposing actions of SREBP-2 and liver-X receptor (LXR) transcription factors function to control cellular cholesterol levels,^90,91^ with growth-promoting factors such as AR and AKT promoting SREBP-2 activity and inhibiting LXR, thereby causing cholesterol accumulation. Statins act by decreasing the production of cholesterol from the liver, thus, lowering its level in blood. At low concentrations, statins exert cardioprotective effects but, at higher concentrations, statins are also capable of tumour suppression by inhibiting small GTPases, which are involved in cellular proliferation, survival, inflammation, metastasis and angiogenesis. Higher statin concentrations are therefore thought to be needed to inhibit the progression of prostate cancer.^92^ Several studies have shown that statin therapy decreases the mortality rate of prostate cancer. Fatostatin (Table 1), which targets a complex of SREBP and the SREBP cleavage-activating protein (SCAP), inhibits lipogenesis and blocks tumour growth and metastasis in autochthonous mouse models of prostate cancer.^93^ Current research suggests the alterations in cholesterol- or geranylgeranyl pyrophosphate-mediated pathways by statins can induce cell death and/or cell cycle arrest. It has been suggested that the effect of statins is mainly mediated through their ability to prevent the accumulation of oncogenic AKT–AR complexes formed on lipid rafts.^92^ A decline in the level of prostate-specific antigen (PSA) is commonly seen upon the use of statins, which seems to indicate that there could be more at play than simply cholesterol-lowering effects.^92^ A detailed study on the use of statins could enhance our understanding of the processes involved in prostate carcinogenesis and potentially identify new therapeutic targets for prostate cancer.

## Amino acid metabolism

The prostate-cancer-specific metabolism of several amino acid has been uncovered over the past 25 years. AR signalling plays a crucial role in coordinating the enhanced uptake of amino acids and also promotes amino acid metabolism. Specific examples of AR-regulated plasma membrane L-type amino acid transporters include LAT1 and LAT3, which coordinate the cellular import of several amino acids including phenylalanine, tryptophan, tyrosine, leucine and arginine.^94^ AR signalling also enhances glutamine uptake and its metabolic assimilation by upregulating the expression of the neutral amino acid transporters ASCT1 and ASCT2. The breakdown of glutamine, the most abundant amino acid in both blood and muscle, provides a source of carbon and nitrogen groups for the TCA cycle and NADPH for the synthesis of nucleotides, proteins and lipids.

The AR-mediated regulation of LAT1 and ASCT2 is of clinical importance as inhibition of these transporters hampers prostate tumour growth^35,95^ and their increased expression provides opportunities for non-invasive diagnostic imaging and disease monitoring in patients with prostate cancer. For example, preclinical studies have demonstrated that the enhanced expression of ASCT2 in response to androgen treatment results in the increased uptake of the synthetic leucine analogue fluciclovine (FACBC) into LNCaP cells.^96,97^ Furthermore, in clinical trials, fluciclovine was not only useful for localising recurrent prostate cancer but also for extra-prostatic metastases.^96^ Ongoing research to define AR-mediated amino acid dependencies in prostate cancer might lead to new prospects in the therapeutic field.

## The Warburg effect

Although most solid tumour cells exhibit some degree of Warburg physiology—that is, they change their source of ATP production from oxidative phosphorylation to aerobic glycolysis—benign prostatic cells follow a different route owing to their markedly different phenotype (the production and secretion of high levels of citrate). Accordingly, early prostate cancer is observed to be dependent on lipids and other energy molecules for ATP production and only at later stages, when the prostate cancer has accumulated multiple mutations, does the Warburg effect become the prominent metabolic route.

Studies of genetically engineered prostate cancer mouse models in conjunction with whole-genome/exome sequencing of human prostate cancer tissue have indicated that activation of PI3K–AKT–mTOR signalling pathways might play a causal role in PTEN-deficiency driven prostate tumorigenesis.^98–100^ Among several genetic aberrations PTEN loss is very common in prostate cancer and accounts for primary (~20%) and castration-resistant (~50%) disease.^101^ Activation of the PI3K–AKT–mTOR pathway is responsible for inducing pyruvate kinase isoenzyme type M-2 (PKM-2), which promotes aerobic glycolysis.^102,103^ On the other hand, glutaminase-2 (GLS-2), induced by p53, has been shown to increase oxidative phosphorylation.^104,105^ *TP53* also suppresses the pentose phosphate pathway by directly interacting with G6PD.^106^ Thus, mutation or loss of *TP53* enhances the Warburg effect and additional anabolic pathways, which, in turn, promote and sustain tumour growth. Hexokinases catalyse the essentially irreversible first step of the glycolytic pathway, and a high level of HK2 expression is associated with poor prognosis in cancer patients.^107,108^ Genetic studies demonstrated that *PTEN* deletion increases HK2 mRNA translation through the activation of the AKT-mTORC1-4EBP1 axis, and *p53* loss enhances HK2 mRNA stability through the inhibition of miR143 biogenesis. Combined deficiency of PTEN and p53 in tumour mouse models synergistically elevates HK2 expression and induces HK2-mediated aerobic glycolysis to fuel aggressive prostate cancer. Together, these results suggest that dysregulation of the PTEN–p53, PI3K–AKT–mTOR pathway drives the Warburg effect to facilitate prostate cancer growth.

## The role of diet and nutrition in prostate cancer management

Epidemiology data suggest that obesity and excessive calorie intake are associated with a higher prostate cancer incidence whereas a low-calorie diet is associated with lower cancer incidence.^109^ A ketogenic diet or a calorie-restricted diet in which overall caloric intake is decreased by 30–40% robustly inhibits the growth of prostate, breast, lung, brain, pancreatic and colorectal cancer cells in preclinical studies,^110^ whereas a high-fat diet is associated with an increased incidence of prostate, breast, pancreatic, colorectal and hepatocellular carcinomas. A diet restricted in specific amino acids such as serine and glycine has been shown to inhibit tumour progression in several mouse models,^111–113^ including prostate cancer. Calorie restriction alone was shown to decrease plasma insulin, and insulin-like growth factor (IGF)-1 levels and increase apoptosis, overall suggesting that calorie-restricted diet may reduce tumour proliferation.^114^ In another human studies, a 15% caloric reduction over 4 years demonstrated a sustained reduction in plasma growth factors and hormones, which have been associated with increased risk of cancer.^114^ The cancer cell location and tissue of origin also contributes to the dietary response. For instance, a ketogenic diet is reported to inhibit tumour growth in brain,^115^ prostate,^116^ pancreatic^117^ and gastric^118^ cancer models, but no effect is seen in colorectal and lung cancer models and, in some instances, a ketogenic diet might even accelerate tumour growth.^119,120^

Several animal studies have concluded that a relationship between nutrient intake and prostate cancer pathogenesis and progression exists, potentially through effects on inflammation, antioxidants and sex hormones. For example, doses of 400 IU or more of vitamin E were seen to result in increased mortality in a study by Miller et al.^121^ However, findings from the Heart Outcomes Prevention Evaluation (HOPE) indicate no association between overall cancer risk and vitamin E intake. Calcium is positively associated with prostate cancer and the risk increases with increased consumption.^122^ Zinc has also been contraindicated in prostate cancer as it appears to promote cancer growth.^123^ Several dietary factors have also been documented; however, trial data exist only for vitamin E, calcium, β-carotene and selenium.^124^ Future studies might focus on uncovering additional gene–nutrient/food interactions and, thus, strengthening the evidence for an association between these parameters, and consequently identifying populations that might benefit from dietary modifications.

## The role of exercise in prostate cancer management

**Fig. 4:**
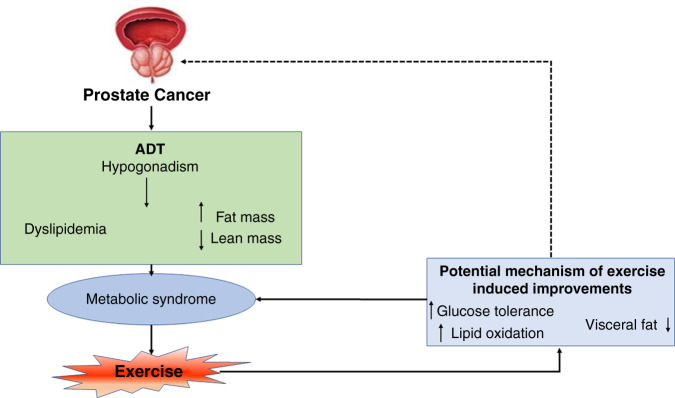
Mechanism of action of exercise in prostate cancer related metabolic syndrome. Potential mechanisms of improvement due to exercise in androgen deprivation therapy (ADT) associated metabolic syndrome. Dashed arrow denotes the inhibitory effect of exercise on prostate cancer progression. Upward arrows denote increased activity whereas downward arrows represent compromised level.

Androgen deprivation therapy (ADT) leads to metabolic syndrome in more than 50% of patients with prostate cancer who are undergoing long-term therapy. Hyperinsulinaemia, hypertension, central obesity, loss of muscle mass and dyslipidaemia are the various components of metabolic syndrome, which can eventually lead to increased cardiovascular disease and mortality.^125^ Exercise has been shown to improve the symptoms of metabolic syndrome (Fig. 4), and exercise interventions, including aerobic, high intensity training and resistance exercises, in patients receiving ADT might alter cardiometabolic risk factors. Consistent exercise has been reported to lead to improvements in muscle mass, strength, physical and psychological wellbeing of these patients.^126^ When cells from the prostate cancer cell line LNCaP were exposed to serum samples taken from individuals before and after exercise (rest serum and exercise serum, respectively), the exercise serum had an inhibitory effect on LNCaP cell growth.^127^ Thus, even short-term exercise had inhibitory effects on cancer cell growth and proliferation. Modest amount of vigorous activity such as biking, jogging, or swimming for ≥3 h a week accompanied by diet tends to increase the survival rate in patients with all prostate cancer.^128^

## Metabolic imaging methods in prostate cancer detection and diagnosis

Imaging techniques such as X-ray, ultrasonography, positron emission tomography (PET), magnetic resonance imaging (MRI) and computed tomography (CT) produce anatomic and functional images of disease. However, anatomic changes are not always visible during the early stages of most cancers including prostate cancer, which limits the value of these imaging methods for early diagnosis.

In addition to morphological differences, cancer cells exhibit increased aerobic glycolysis and, accordingly, although this characteristic is not demonstrated by all cancer types, rapidly growing tumours commonly demonstrate uptake of FDG on PET scans.^129^ In the proper setting, this increased glucose uptake can indicate that the ‘glycolytic switch’ is turned on, and FDG-PET scanning has become a routine procedure for diagnosis, initial staging, and assessment of therapy response for many cancer types. Furthermore, studies have also associated higher primary tumour FDG uptake with worse prognosis.^129^ However, the uptake of FDG is low in most primary prostate cancers due to relatively low glucose metabolism. Besides this, FDG does not undergo for further metabolism and remains trapped in the cell^130^ leading to its accumulation in the bladder which often makes it impossible to detect prostate cancer on PET scans. A low uptake of FDG in primary prostate cancer might indicate the requirement of metabolic substrates other than glucose to meet the anabolic demands which could hold utility for clinical imaging. For example, another PET agent that has had some success is ^11^C acetate, which is converted to acetyl coenzyme-A in mitochondria, followed by its rapid clearance as carbon dioxide through the TCA cycle. Yashimoto et al.^131^ successfully demonstrated a higher accumulation of acetate in tumour cell lines compared with a fibroblast cell line.^131^ This difference is due to enhanced lipid synthesis in tumour cells. Given its crucial role in cell membrane formation, acetate could be used a probe for this anabolic pathway in cancer tissue. Another metabolic PET agent is based on choline, which is considered to be an excellent biomarker of proliferating cancer cells. Alterations in choline metabolism are common among many cancer cell types and are thought to reflect the increased demands of proliferating cancer cells. Both choline and acetate can be used for imaging prostate cancer with PET and have been reported to be more sensitive than FDG-PET for detecting primary lesions, regional lymph node metastasis and bone metastasis. However, these agents are often labelled with ^11^C, which, despite its relatively good imaging properties, has a short half-life (20 min), thereby limiting its use to imaging centres with cyclotrons and radiochemistry facilities.

A new molecular imaging technique, hyperpolarised carbon-13 (^13^C) MRI, has been introduced to monitor the uptake and metabolism of endogenous biomolecules. ^13^C-MRI is particularly attractive because carbon serves as backbone for nearly all organic molecules, thus allowing the investigation of a wide range of biochemical processes that are relevant to human diseases. Although the low natural abundance of the ^13^C isotope (1.1%) has made in vivo imaging extremely challenging, this limitation has been overcome by the development of the dynamic nuclear polarisation technique, which can temporarily increase the signal of ^13^C-labelled molecules by more than 10,000-fold. Studies of ^13^C-pyruvate after hyperpolarisation reveal its conversion to ^13^C-lactate, which can be readily monitored using magnetic resonance (MR) spectroscopy methods. Preclinical models of prostate cancer have shown an increased signal from hyperpolarised lactate relative to normal tissues due to increased aerobic glycolysis within the tumour.^132^ Studies performed by Nelson et al.^30^ revealed an increased flux of [1-^13^C] pyruvate to [1-^13^C] lactate in prostate cancer patients.^30^ This first-in-human study demonstrated that the method works in humans and could provide an opportunity for detecting and staging cancer, as well as monitoring tumour progression and response to therapy. However, the use of hyperpolarised MRI technology in human patients is challenging as it requires specialised instrumentation, label preparation in a clean environment, and rapid delivery to patients—the half-life of hyperpolarised ^13^C-pyruvate is only a few minutes.

## Conclusions and future perspectives

Over the past decade, considerable progress has been made towards understanding the basic mechanisms and biological consequences that are associated with metabolic reprogramming in cancer. Metabolic reprogramming is essential for the biology of malignant cells—it supports the ability of cancer cells to survive and grow by adapting to local conditions. Metabolic reprogramming is a consequence of the overexpression of oncogenes such as *KRAS* and the deficiency of tumour suppressor genes such as *TP53*, which result in the activation of master regulators of metabolism such as PI3K, mTOR-1 and other signalling pathways, including transcriptional networks involving HIFs, MYC and SREBPs. However, metabolism is also influenced by physical aspects of the TME, including hypoxia, vascularity and tissue context. Early discoveries enabled conceptual advances in the study of tumour metabolism, which occurred in parallel with advances in genetics, cell biology and spectroscopy. Modern approaches to studying metabolism, including imaging studies, have redefined, and overturned, several notions, including the ubiquity of Warburg physiology in cancer. Even so, the spirit of Warburg’s hypothesis still holds true in many cases, as aerobic glycolysis often differentiates tumours from their normal counterparts and enables their growth and proliferation. Prostate cancer has peculiar metabolic properties, probably due to the unique metabolic characteristics of normal prostate tissue. For example, unlike normal prostate tissue, in which the TCA cycle is inhibited, in the early stages of prostate cancers the TCA cycle is activated, resulting in an increased dependence on oxidative phosphorylation, anaplerotic metabolism and lipogenesis. Several of these metabolic changes are probably orchestrated by AR, fuelling experimental efforts to dissect AR-mediated metabolic dependencies in prostate tumours. Overall, these more-recent efforts have shaped new functional metabolic imaging approaches and translated the development of novel metabolic inhibitors (Table 1) for prostate cancer diagnosis and treatment.

Our current understanding of the complexity of tumour metabolism and the molecular pathways involved is rapidly evolving, and fundamental principles of tumour metabolism continue to be redefined. Despite extensive research in cancer metabolism, however, several questions remain unanswered. What is the driving force that leads to metabolic dependencies? What specific molecular interactions can be targeted for therapeutic exploitation? Instead of targeting tumour cells, could the TME be targeted? The TME, through its nutrient gradients, pH and proximity to blood vessels, undoubtedly influences regional metabolic fluxes throughout the tumours. What are the roles of diet, exercise and pharmacotherapy in modifying the microenvironment of tumour cells?

Some tumour cell metabolic vulnerabilities observed in vivo are not observed in cultured cell models. Moreover, even metabolic phenotypes are not consistent across single tumours among patients. Little is known about metabolic pathways in non-proliferating tumour cells (circulating tumour cells). A better understanding of cancer metabolism, including the molecular basis of adaptation, resistance, and the ability to thrive in a changing microenvironment, is essential for uncovering new potential avenues for treatment and will be critical for the successful translation of targeted metabolic inhibitors and diagnostic imaging approaches. The infusion of ^13^C-labelled substrates into patients with lung^37,133^ or kidney^134^ tumours has been highly informative, and similar efforts are warranted in men with prostate cancer. Prostate cancer cells preferentially metastasise to distant sites such as bone that are metabolically optimised to support their growth. Research into the metabolic dependencies of the seed and those of the soil might lead to new metabolic interventions to prevent metastatic dissemination of prostate tumours, a process that currently renders this disease incurable.

## Data Availability

Not applicable.

## References

[1] DeBerardinis, R. J. & Thompson, C. B. Cellular metabolism and disease: what do metabolic outliers teach us? *Cell* **148**, 1132–1144 (2012). 10.1016/j.cell.2012.02.032 PMC3337773 22424225

[2] Pavlova NN, Thompson CB (2016). The emerging hallmarks of cancer metabolism. Cell Metab..

[3] Warburg O, Wind F, Negelein E (1927). The metabolism of tumors in the body. J. Gen. Physiol..

[4] Vander Heiden MG, Cantley LC, Thompson CB (2009). Understanding the Warburg effect: the metabolic requirements of cell proliferation. Science.

[5] Malumbres M, Barbacid M (2003). RAS oncogenes: the first 30 years. Nat. Rev. Cancer.

[6] Newsholme EA, Crabtree B, Ardawi MS (1985). The role of high rates of glycolysis and glutamine utilization in rapidly dividing cells. Biosci. Rep..

[7] Nakao, K., Minato, N. & Uemoto, S. (eds). *Innovative Medicine*: *Basic Research and Development*: Springer; (2015). 29787043

[8] DeBerardinis RJ, Lum JJ, Hatzivassiliou G, Thompson CB (2008). The biology of cancer: metabolic reprogramming fuels cell growth and proliferation. Cell Metab..

[9] DeBerardinis RJ, Chandel NS (2016). Fundamentals of cancer metabolism. Sci. Adv..

[10] Duvel K, Yecies JL, Menon S, Raman P, Lipovsky AI, Souza AL (2010). Activation of a metabolic gene regulatory network downstream of mTOR complex 1. Mol. Cell.

[11] Barthel A, Okino ST, Liao J, Nakatani K, Li J, Whitlock JP (1999). Regulation of GLUT1 gene transcription by the serine/threonine kinase Akt1. J. Biol. Chem..

[12] Lee G, Zheng Y, Cho S, Jang C, England C, Dempsey JM (2017). Post-transcriptional regulation of de novo lipogenesis by mTORC1-S6K1-SRPK2 signaling. Cell.

[13] Bauer DE, Hatzivassiliou G, Zhao F, Andreadis C, Thompson CB (2005). ATP citrate lyase is an important component of cell growth and transformation. Oncogene.

[14] Wang W, Fridman A, Blackledge W, Connelly S, Wilson IA, Pilz RB (2009). The phosphatidylinositol 3-kinase/akt cassette regulates purine nucleotide synthesis. J. Biol. Chem..

[15] Guo D, Prins RM, Dang J, Kuga D, Iwanami A, Soto H (2009). EGFR signaling through an Akt-SREBP-1-dependent, rapamycin-resistant pathway sensitizes glioblastomas to antilipogenic therapy. Sci. Signal..

[16] Guo D, Reinitz F, Youssef M, Hong C, Nathanson D, Akhavan D (2011). An LXR agonist promotes glioblastoma cell death through inhibition of an EGFR/AKT/SREBP-1/LDLR-dependent pathway. Cancer Discov..

[17] Villa GR, Hulce JJ, Zanca C, Bi J, Ikegami S, Cahill GL (2016). An LXR-cholesterol axis creates a metabolic co-dependency for brain cancers. Cancer Cell.

[18] Sun L, Song L, Wan Q, Wu G, Li X, Wang Y (2015). cMyc-mediated activation of serine biosynthesis pathway is critical for cancer progression under nutrient deprivation conditions. Cell Res..

[19] Nikiforov MA, Chandriani S, O’Connell B, Petrenko O, Kotenko I, Beavis A (2002). A functional screen for Myc-responsive genes reveals serine hydroxymethyltransferase, a major source of the one-carbon unit for cell metabolism. Mol. Cell Biol..

[20] Ben-Sahra I, Howell JJ, Asara JM, Manning BD (2013). Stimulation of de novo pyrimidine synthesis by growth signaling through mTOR and S6K1. Science.

[21] Robitaille AM, Christen S, Shimobayashi M, Cornu M, Fava LL, Moes S (2013). Quantitative phosphoproteomics reveal mTORC1 activates de novo pyrimidine synthesis. Science.

[22] Hanahan D, Weinberg RA (2000). The hallmarks of cancer. Cell.

[23] Efeyan A, Comb WC, Sabatini DM (2015). Nutrient-sensing mechanisms and pathways. Nature.

[24] Chen S, Sang N (2016). Hypoxia-inducible factor-1: a critical player in the survival strategy of stressed cells. J. Cell Biochem..

[25] Corcoran SE, O’Neill LA (2016). HIF1alpha and metabolic reprogramming in inflammation. J. Clin. Invest..

[26] Gonzalez A, Hall MN (2017). Nutrient sensing and TOR signaling in yeast and mammals. EMBO J..

[27] Nishida N, Yano H, Nishida T, Kamura T, Kojiro M (2006). Angiogenesis in cancer. Vasc. Health Risk Manag..

[28] Liu IJ, Zafar MB, Lai YH, Segall GM, Terris MK (2001). Fluorodeoxyglucose positron emission tomography studies in diagnosis and staging of clinically organ-confined prostate cancer. Urology.

[29] Vavere AL, Kridel SJ, Wheeler FB, Lewis JS (2008). 1-11C-acetate as a PET radiopharmaceutical for imaging fatty acid synthase expression in prostate cancer. J. Nucl. Med..

[30] Nelson, S. J., Kurhanewicz, J., Vigneron, D. B., Larson, P. E., Harzstark, A. L., Ferrone, M. et al. Metabolic imaging of patients with prostate cancer using hyperpolarized [1-(1)(3)C]pyruvate. *Sci. Trans.l Med*. **5**, 198ra108 (2013). 10.1126/scitranslmed.3006070 PMC4201045 23946197

[31] Thomlinson RH (1977). Hypoxia and tumours. J. Clin. Pathol. Suppl..

[32] Semenza GL (2003). Targeting HIF-1 for cancer therapy. Nat. Rev. Cancer.

[33] Semenza GL (2013). HIF-1 mediates metabolic responses to intratumoral hypoxia and oncogenic mutations. J. Clin. Invest..

[34] Krock BL, Skuli N, Simon MC (2011). Hypoxia-induced angiogenesis: good and evil. Genes Cancer.

[35] Wang Q, Bailey CG, Ng C, Tiffen J, Thoeng A, Minhas V (2011). Androgen receptor and nutrient signaling pathways coordinate the demand for increased amino acid transport during prostate cancer progression. Cancer Res..

[36] Gaude E, Frezza C (2016). Tissue-specific and convergent metabolic transformation of cancer correlates with metastatic potential and patient survival. Nat. Commun..

[37] Hensley CT, Faubert B, Yuan Q, Lev-Cohain N, Jin E, Kim J (2016). Metabolic heterogeneity in human lung tumors. Cell.

[38] Dupuy F, Tabaries S, Andrzejewski S, Dong Z, Blagih J, Annis MG (2015). PDK1-dependent metabolic reprogramming dictates metastatic potential in breast cancer. Cell Metab.

[39] Bader DA, Hartig SM, Putluri V, Foley C, Hamilton MP, Smith EA (2019). Mitochondrial pyruvate import is a metabolic vulnerability in androgen receptor-driven prostate cancer. Nat. Metab..

[40] Verze P, Cai T, Lorenzetti S (2016). The role of the prostate in male fertility, health and disease. Nat. Rev. Urol..

[41] Barron ES, Huggins C (1946). The metabolism of the prostate; transamination and citric acid. J. Urol..

[42] Flavin R, Zadra G, Loda M (2011). Metabolic alterations and targeted therapies in prostate cancer. J. Pathol.

[43] Czernin J, Benz MR, Allen-Auerbach MS (2009). PET imaging of prostate cancer using C-acetate. PET Clin..

[44] Strmiska V, Michalek P, Eckschlager T, Stiborova M, Adam V, Krizkova S (2019). Prostate cancer-specific hallmarks of amino acids metabolism: towards a paradigm of precision medicine. Biochim. Biophys. Acta Rev. Cancer.

[45] Prescott JL, Blok L, Tindall DJ (1998). Isolation and androgen regulation of the human homeobox cDNA, NKX3.1. Prostate.

[46] Costello LC, Liu Y, Zou J, Franklin RB (1999). Evidence for a zinc uptake transporter in human prostate cancer cells which is regulated by prolactin and testosterone. J. Biol. Chem.

[47] Lao L, Franklin RB, Costello LC (1993). High-affinity L-aspartate transporter in prostate epithelial cells that is regulated by testosterone. Prostate.

[48] Franklin RB, Milon B, Feng P, Costello LC (2005). Zinc and zinc transporters in normal prostate and the pathogenesis of prostate cancer. Front. Biosci..

[49] Johnson LA, Kanak MA, Kajdacsy-Balla A, Pestaner JP, Bagasra O (2010). Differential zinc accumulation and expression of human zinc transporter 1 (hZIP1) in prostate glands. Methods.

[50] Swinnen JV, Van Veldhoven PP, Esquenet M, Heyns W, Verhoeven G (1996). Androgens markedly stimulate the accumulation of neutral lipids in the human prostatic adenocarcinoma cell line LNCaP. Endocrinology.

[51] Audet-Walsh E, Yee T, McGuirk S, Vernier M, Ouellet C, St-Pierre J (2017). Androgen-dependent repression of ERRgamma reprograms metabolism in prostate cancer. Cancer Res.

[52] Tsouko E, Khan AS, White MA, Han JJ, Shi Y, Merchant FA (2014). Regulation of the pentose phosphate pathway by an androgen receptor-mTOR-mediated mechanism and its role in prostate cancer cell growth. Oncogenesis.

[53] Patra KC, Hay N (2014). The pentose phosphate pathway and cancer. Trends Biochem. Sci..

[54] Huggins C, Hodges CV (1972). Studies on prostatic cancer. I. The effect of castration, of estrogen and androgen injection on serum phosphatases in metastatic carcinoma of the prostate. CA Cancer J. Clin..

[55] Payne H, Mason M (2011). Androgen deprivation therapy as adjuvant/neoadjuvant to radiotherapy for high-risk localised and locally advanced prostate cancer: recent developments. Br. J. Cancer.

[56] Ather MH, Abbas F, Faruqui N, Israr M, Pervez S (2008). Correlation of three immunohistochemically detected markers of neuroendocrine differentiation with clinical predictors of disease progression in prostate cancer. BMC Urol.

[57] Choi YJ, Lin CP, Ho JJ, He X, Okada N, Bu P (2011). miR-34 miRNAs provide a barrier for somatic cell reprogramming. Nat. Cell Biol..

[58] Kareta MS, Gorges LL, Hafeez S, Benayoun BA, Marro S, Zmoos AF (2015). Inhibition of pluripotency networks by the Rb tumor suppressor restricts reprogramming and tumorigenesis. Cell Stem Cell.

[59] Schvartzman JM, Thompson CB, Finley LWS (2018). Metabolic regulation of chromatin modifications and gene expression. J. Cell Biol..

[60] Wang HJ, Pochampalli M, Wang LY, Zou JX, Li PS, Hsu SC (2019). KDM8/JMJD5 as a dual coactivator of AR and PKM2 integrates AR/EZH2 network and tumor metabolism in CRPC. Oncogene.

[61] Reina-Campos M, Linares JF, Duran A, Cordes T, L’Hermitte A, Badur MG (2019). Increased serine and one-carbon pathway metabolism by PKClambda/iota deficiency promotes neuroendocrine prostate cancer. Cancer Cell.

[62] Choi YK, Park KG (2018). Targeting glutamine metabolism for cancer treatment. Biomol. Ther. (Seoul).

[63] Choi, S. Y. C., Ettinger, S. L., Lin, D., Xue, H., Ci, X., Nabavi, N. et al. Targeting MCT4 to reduce lactic acid secretion and glycolysis for treatment of neuroendocrine prostate cancer. *Cancer Med*. **7**(7), 3385–3392 (2018). 10.1002/cam4.1587 PMC6051138 29905005

[64] Bader DA, McGuire SE (2020). Tumour metabolism and its unique properties in prostate adenocarcinoma. Nat. Rev. Urol..

[65] Latonen L, Afyounian E, Jylha A, Nattinen J, Aapola U, Annala M (2018). Integrative proteomics in prostate cancer uncovers robustness against genomic and transcriptomic aberrations during disease progression. Nat. Commun..

[66] Shao Y, Ye G, Ren S, Piao HL, Zhao X, Lu X (2018). Metabolomics and transcriptomics profiles reveal the dysregulation of the tricarboxylic acid cycle and related mechanisms in prostate cancer. Int. J. Cancer.

[67] Costello LC, Franklin RB (2006). The clinical relevance of the metabolism of prostate cancer; zinc and tumor suppression: connecting the dots. Mol. Cancer.

[68] Franklin RB, Zou J, Yu Z, Costello LC (2006). EAAC1 is expressed in rat and human prostate epithelial cells; functions as a high-affinity L-aspartate transporter; and is regulated by prolactin and testosterone. BMC Biochem.

[69] Heinz S, Freyberger A, Lawrenz B, Schladt L, Schmuck G, Ellinger-Ziegelbauer H (2017). Mechanistic investigations of the mitochondrial complex I inhibitor rotenone in the context of pharmacological and safety evaluation. Sci. Rep..

[70] Wheaton WW, Weinberg SE, Hamanaka RB, Soberanes S, Sullivan LB, Anso E (2014). Metformin inhibits mitochondrial complex I of cancer cells to reduce tumorigenesis. elife.

[71] Zaidi S, Gandhi J, Joshi G, Smith NL, Khan SA (2019). The anticancer potential of metformin on prostate cancer. Prostate Cancer Prostatic Dis..

[72] Naguib A, Mathew G, Reczek CR, Watrud K, Ambrico A, Herzka T (2018). Mitochondrial complex I inhibitors expose a vulnerability for selective killing of Pten-null cells. Cell Rep..

[73] Zaidi N, Lupien L, Kuemmerle NB, Kinlaw WB, Swinnen JV, Smans K (2013). Lipogenesis and lipolysis: the pathways exploited by the cancer cells to acquire fatty acids. Prog. Lipid Res..

[74] Clarke NW, Brown MD (2007). The influence of lipid metabolism on prostate cancer development and progression: is it time for a closer look?. Eur. Urol..

[75] Suburu J, Chen YQ (2012). Lipids and prostate cancer. Prostaglandins Other Lipid Mediat..

[76] Yue S, Li J, Lee SY, Lee HJ, Shao T, Song B (2014). Cholesteryl ester accumulation induced by PTEN loss and PI3K/AKT activation underlies human prostate cancer aggressiveness. Cell Metab..

[77] Gazi E, Gardner P, Lockyer NP, Hart CA, Brown MD, Clarke NW (2007). Direct evidence of lipid translocation between adipocytes and prostate cancer cells with imaging FTIR microspectroscopy. J. Lipid Res..

[78] Swinnen JV, Roskams T, Joniau S, Van Poppel H, Oyen R, Baert L (2002). Overexpression of fatty acid synthase is an early and common event in the development of prostate cancer. Int. J. Cancer.

[79] Liu Y (2006). Fatty acid oxidation is a dominant bioenergetic pathway in prostate cancer. Prostate Cancer Prostatic Dis..

[80] Lloyd MD, Yevglevskis M, Lee GL, Wood PJ, Threadgill MD, Woodman T (2013). J. alpha-Methylacyl-CoA racemase (AMACR): metabolic enzyme, drug metabolizer and cancer marker P504S. Prog. Lipid Res..

[81] Zha S, Ferdinandusse S, Denis S, Wanders RJ, Ewing CM, Luo J (2003). Alpha-methylacyl-CoA racemase as an androgen-independent growth modifier in prostate cancer. Cancer Res..

[82] Ahmad F, Patrick S, Sheikh T, Sharma V, Pathak P, Malgulwar PB (2017). Telomerase reverse transcriptase (TERT)–enhancer of zeste homolog 2 (EZH2) network regulates lipid metabolism and DNA damage responses in glioblastoma. J. Neurochem..

[83] Van de Sande, T., Roskams, T., Lerut, E., Joniau, S, Van Poppel, H., Verhoeven, G. et al. High-level expression of fatty acid synthase in human prostate cancer tissues is linked to activation and nuclear localization of Akt/PKB. *J. Pathol*. **206**, 214–219 (2005). 10.1002/path.1760 15880754

[84] Rhodes DR, Yu J, Shanker K, Deshpande N, Varambally R, Ghosh D (2004). ONCOMINE: a cancer microarray database and integrated data-mining platform. Neoplasia.

[85] Guo D, Bell EH, Mischel P, Chakravarti A (2014). Targeting SREBP-1-driven lipid metabolism to treat cancer. Curr. Pharm. Des..

[86] Zadra G, Ribeiro CF, Chetta P, Ho Y, Cacciatore S, Gao X (2019). Inhibition of de novo lipogenesis targets androgen receptor signaling in castration-resistant prostate cancer. Proc. Natl Acad. Sci. USA.

[87] O’Malley BW (1971). Mechanisms of action of steroid hormones. N. Engl. J. Med..

[88] Ayyagari VN, Wang X, Diaz-Sylvester PL, Groesch K, Brard L (2020). Assessment of acyl-CoA cholesterol acyltransferase (ACAT-1) role in ovarian cancer progression—an in vitro study. PLoS ONE.

[89] Wu X, Daniels G, Lee P, Monaco ME (2014). Lipid metabolism in prostate cancer. Am. J. Clin. Exp. Urol..

[90] Patel D, Ahmad F, Kambach DM, Sun Q, Halim AS, Kramp T (2019). LXRbeta controls glioblastoma cell growth, lipid balance, and immune modulation independently of ABCA1. Sci. Rep..

[91] Ahmad, F., Sun, Q., Patel, D. & Stommel, J. M. Cholesterol metabolism: a potential therapeutic target in glioblastoma. *Cancers (Basel)* **11**, (2019). 10.3390/cancers11020146. 10.3390/cancers11020146 PMC6406281 30691162

[92] Roy M, Kung HJ, Ghosh PM (2011). Statins and prostate cancer: role of cholesterol inhibition vs. prevention of small GTP-binding proteins. Am. J. Cancer Res..

[93] Svensson RU, Parker SJ, Eichner LJ, Kolar MJ, Wallace M, Brun SN (2016). Inhibition of acetyl-CoA carboxylase suppresses fatty acid synthesis and tumor growth of non-small-cell lung cancer in preclinical models. Nat. Med..

[94] Wang Q, Tiffen J, Bailey CG, Lehman ML, Ritchie W, Fazli L (2013). Targeting amino acid transport in metastatic castration-resistant prostate cancer: effects on cell cycle, cell growth, and tumor development. J. Natl Cancer Inst..

[95] Wang Q, Hardie RA, Hoy AJ, van Geldermalsen M, Gao D, Fazli L (2015). Targeting ASCT2-mediated glutamine uptake blocks prostate cancer growth and tumour development. J. Pathol..

[96] Savir-Baruch B, Zanoni L, Schuster DM (2018). Imaging of prostate cancer using fluciclovine. Urol. Clin. North Am..

[97] Okudaira H, Oka S, Ono M, Nakanishi T, Schuster DM, Kobayashi M (2014). Accumulation of trans-1-amino-3-[(18)F]fluorocyclobutanecarboxylic acid in prostate cancer due to androgen-induced expression of amino acid transporters. Mol. Imaging Biol..

[98] Barbieri CE, Baca SC, Lawrence MS, Demichelis F, Blattner M, Theurillat JP (2012). Exome sequencing identifies recurrent SPOP, FOXA1 and MED12 mutations in prostate cancer. Nat. Genet..

[99] Chen ML, Xu PZ, Peng XD, Chen WS, Guzman G, Yang X (2006). The deficiency of Akt1 is sufficient to suppress tumor development in Pten+/− mice. Genes Dev..

[100] Grasso CS, Wu YM, Robinson DR, Cao X, Dhanasekaran SM, Khan AP (2012). The mutational landscape of lethal castration-resistant prostate cancer. Nature.

[101] Jamaspishvili T, Berman DM, Ross AE, Scher HI, De Marzo AM, Squire JA (2018). Clinical implications of PTEN loss in prostate cancer. Nat. Rev. Urol..

[102] Fang M, Shen Z, Huang S, Zhao L, Chen S, Mak TW (2010). The ER UDPase ENTPD5 promotes protein N-glycosylation, the Warburg effect, and proliferation in the PTEN pathway. Cell.

[103] Sun Q, Chen X, Ma J, Peng H, Wang F, Zha X (2011). Mammalian target of rapamycin up-regulation of pyruvate kinase isoenzyme type M2 is critical for aerobic glycolysis and tumor growth. Proc. Natl Acad. Sci. USA.

[104] Hu W, Zhang C, Wu R, Sun Y, Levine A, Feng Z (2010). Glutaminase 2, a novel p53 target gene regulating energy metabolism and antioxidant function. Proc. Natl Acad. Sci. USA.

[105] Suzuki S, Tanaka T, Poyurovsky MV, Nagano H, Mayama T, Ohkubo S (2010). Phosphate-activated glutaminase (GLS2), a p53-inducible regulator of glutamine metabolism and reactive oxygen species. Proc. Natl Acad. Sci. USA.

[106] Jiang P, Du W, Wang X, Mancuso A, Gao X, Wu M (2011). p53 regulates biosynthesis through direct inactivation of glucose-6-phosphate dehydrogenase. Nat. Cell Biol..

[107] Mathupala SP, Rempel A, Pedersen PL (2001). Glucose catabolism in cancer cells: identification and characterization of a marked activation response of the type II hexokinase gene to hypoxic conditions. J. Biol. Chem..

[108] Wolf A, Agnihotri S, Micallef J, Mukherjee J, Sabha N, Cairns R (2011). Hexokinase 2 is a key mediator of aerobic glycolysis and promotes tumor growth in human glioblastoma multiforme. J. Exp. Med..

[109] Rawla P (2019). Epidemiology of prostate cancer. World J. Oncol..

[110] Weber DD, Aminzadeh-Gohari S, Tulipan J, Catalano L, Feichtinger RG, Kofler B (2020). Ketogenic diet in the treatment of cancer—-where do we stand?. Mol. Metab..

[111] Maddocks ODK, Athineos D, Cheung EC, Lee P, Zhang T, van den Broek NJF (2017). Modulating the therapeutic response of tumours to dietary serine and glycine starvation. Nature.

[112] Sullivan LB, Gui DY, Hosios AM, Bush LN, Freinkman E, Vander Heiden MG (2015). Supporting aspartate biosynthesis is an essential function of respiration in proliferating cells. Cell.

[113] Hoffman RM (2019). Clinical studies of methionine-restricted diets for cancer patients. Methods Mol. Biol..

[114] Gray A, Dang BN, Moore TB, Clemens R, Pressman P (2020). A review of nutrition and dietary interventions in oncology. SAGE Open Med..

[115] Abdelwahab MG, Fenton KE, Preul MC, Rho JM, Lynch A, Stafford P (2012). The ketogenic diet is an effective adjuvant to radiation therapy for the treatment of malignant glioma. PLoS ONE.

[116] Freedland SJ, Mavropoulos J, Wang A, Darshan M, Demark-Wahnefried W, Aronson WJ (2008). Carbohydrate restriction, prostate cancer growth, and the insulin-like growth factor axis. Prostate.

[117] Wheatley KE, Williams EA, Smith NC, Dillard A, Park EY, Nunez NP (2008). Low-carbohydrate diet versus caloric restriction: effects on weight loss, hormones, and colon tumor growth in obese mice. Nutr. Cancer.

[118] Otto C, Kaemmerer U, Illert B, Muehling B, Pfetzer N, Wittig R (2008). Growth of human gastric cancer cells in nude mice is delayed by a ketogenic diet supplemented with omega-3 fatty acids and medium-chain triglycerides. BMC Cancer.

[119] Liskiewicz AD, Kasprowska D, Wojakowska A, Polanski K, Lewin-Kowalik J, Kotulska K (2016). Long-term high fat ketogenic diet promotes renal tumor growth in a rat model of tuberous sclerosis. Sci. Rep..

[120] Kang HB, Fan J, Lin R, Elf S, Ji Q, Zhao L (2015). Metabolic rewiring by oncogenic BRAF V600E links ketogenesis pathway to BRAF-MEK1 signaling. Mol. Cell.

[121] Miller ER, Pastor-Barriuso R, Dalal D, Riemersma RA, Appel LJ, Guallar E (2005). Meta-analysis: high-dosage vitamin E supplementation may increase all-cause mortality. Ann. Intern. Med..

[122] Butler LM, Wong AS, Koh WP, Wang R, Yuan JM, Yu MC (2010). Calcium intake increases risk of prostate cancer among Singapore Chinese. Cancer Res..

[123] Leitzmann MF, Stampfer MJ, Wu K, Colditz GA, Willett WC, Giovannucci EL (2003). Zinc supplement use and risk of prostate cancer. J. Natl Cancer Inst..

[124] Sonn GA, Aronson W, Litwin MS (2005). Impact of diet on prostate cancer: a review. Prostate Cancer Prostatic Dis..

[125] Kiwata JL, Dorff TB, Schroeder ET, Gross ME, Dieli-Conwright CM (2016). A review of clinical effects associated with metabolic syndrome and exercise in prostate cancer patients. Prostate Cancer Prostatic Dis..

[126] Lee IM (2003). Physical activity and cancer prevention-data from epidemiologic studies. Med. Sci. Sports Exerc..

[127] Rundqvist H, Augsten M, Stromberg A, Rullman E, Mijwel S, Kharaziha P (2013). Effect of acute exercise on prostate cancer cell growth. PLoS ONE.

[128] Kenfield SA, Stampfer MJ, Giovannucci E, Chan JM (2011). Physical activity and survival after prostate cancer diagnosis in the health professionals follow-up study. J. Clin. Oncol..

[129] Chung HW, Lee EJ, Cho YH, Yoon SY, So Y, Kim SY (2010). High FDG uptake in PET/CT predicts worse prognosis in patients with metastatic gastric adenocarcinoma. J. Cancer Res. Clin. Oncol..

[130] Gallagher BM, Fowler JS, Gutterson NI, MacGregor RR, Wan CN, Wolf AP (1978). Metabolic trapping as a principle of oradiopharmaceutical design: some factors resposible for the biodistribution of [18F] 2-deoxy-2-fluoro-D-glucose. J. Nucl. Med..

[131] Yoshimoto M, Waki A, Obata A, Furukawa T, Yonekura Y, Fujibayashi Y (2004). Radiolabeled choline as a proliferation marker: comparison with radiolabeled acetate. Nucl. Med. Biol..

[132] Albers MJ, Bok R, Chen AP, Cunningham CH, Zierhut ML, Zhang VY (2008). Hyperpolarized 13C lactate, pyruvate, and alanine: noninvasive biomarkers for prostate cancer detection and grading. Cancer Res..

[133] Faubert B, Li KY, Cai L, Hensley CT, Kim J, Zacharias LG (2017). Lactate metabolism in human lung tumors. Cell.

[134] Courtney KD, Bezwada D, Mashimo T, Pichumani K, Vemireddy V, Funk AM (2018). Isotope tracing of human clear cell renal cell carcinomas demonstrates suppressed glucose oxidation in vivo. Cell Metab.

[135] Richards KA, Liou JI, Cryns VL, Downs TM, Abel EJ, Jarrard DF (2018). Metformin use is associated with improved survival for patients with advanced prostate cancer on androgen deprivation therapy. J. Urol..

[136] Vancura A, Bu P, Bhagwat M, Zeng J, Vancurova I (2018). Metformin as an anticancer agent. Trends Pharmacol. Sci..

[137] Liu X, Romero IL, Litchfield LM, Lengyel E, Locasale JW (2016). Metformin targets central carbon metabolism and reveals mitochondrial requirements in human cancers. Cell Metab..

[138] Molina JR, Sun Y, Protopopova M, Gera S, Bandi M, Bristow C (2018). An inhibitor of oxidative phosphorylation exploits cancer vulnerability. Nat. Med..

[139] Zadra G, Photopoulos C, Tyekucheva S, Heidari P, Weng QP, Fedele G (2014). A novel direct activator of AMPK inhibits prostate cancer growth by blocking lipogenesis. EMBO Mol. Med..

